# Stress, health and lifestyle behaviours during COVID-19 home confinement in portuguese adults

**DOI:** 10.1192/j.eurpsy.2021.831

**Published:** 2021-08-13

**Authors:** A.P. Amaral, J. Figueiredo, A. Ferreira, S. Seco, A. Loureiro, L.S. Costa

**Affiliations:** 1 Coimbra Health School, Polytechnic of Coimbra, Coimbra, Portugal; 2 Occupational And Environmental Health, Polytechnic of Coimbra, Coimbra, Portugal

**Keywords:** stress, health, lifestyle, COVID-19

## Abstract

**Introduction:**

The confinement associated with COVID-19 pandemic was an experience with significant physical and mental health implications, including higher stress levels, decreased sleep quality, pain symptoms and changes in lifestyle behaviours.

**Objectives:**

The main goal of this study was to analyze the relationship between stress and health variables (sleep, health symptoms, health perception, and lifestyle behaviours) in a Portuguese university during COVID-19 home confinement.

**Methods:**

A cross-sectional online survey design was conducted. A sample of 263 Portuguese workers (64.3% females), with mean age of 48.3 years (sd=8.9), filled in the PSS10, answering questions concerning health symptoms (perceived health, pain symptoms and fatigue), lifestyle behaviours (sleep and eating habits, use of alcohol and tobacco) during COVID-19 home confinement. A descriptive statistical analysis, a Pearson correlation analyses and the t Student test, for independent samples, were performed.

**Results:**

The results showed significant correlations between stress and perceived health (r=-.404; p<.0001), arms pain (r=.212; p=.002), legs pain (r=.201; p=.003), back pain (r=.219; p=.001), headache (r=.289; p<.0001) and fatigue (r=.295; p<.0001). Concerning lifestyle behaviours, the results showed significant correlations between stress and sleep (r=-.552; p<.0001) and stress is significantly higher (p<.0001) in individuals who have changed their eating habits.

**Conclusions:**

During the COVID-19 home confinement, higher stress levels are associated with a worse perception of health, more pain symptoms (legs, arms, back, headache), worse sleep quality and more fatigue. Moreover, the individuals with higher levels of stress have changed their eating habits. Lastly, health promotion interventions are needed in order to minimize the impact of home confinement in health.

